# Association between the Consumption of Ultra-Processed Foods and Asthma in Adults from Ribeirão Preto, São Paulo, Brazil

**DOI:** 10.3390/nu15143165

**Published:** 2023-07-17

**Authors:** Hellen Cristina Oliveira Amorim Serra, Lívia Carolina Sobrinho Rudakoff, Alessandra Karla Oliveira Amorim Muniz, Elma Izze da Silva Magalhães, Maylla Luanna Barbosa Martins Bragança, Antônio Augusto Moura da Silva, Elcio dos Santos Oliveira Vianna, Heloisa Bettiol, Marco Antonio Barbieri

**Affiliations:** 1 Postgraduate Programme in Collective Health, Federal University of Maranhão, São Luís 65020-070, Brazil; livia.rudakoff@ifma.edu.br (L.C.S.R.); alessandrakoa.amorim@gmail.com (A.K.O.A.M.); elmaizzenutri@gmail.com (E.I.d.S.M.); mayllabmartins@gmail.com (M.L.B.M.B.); aamouradasilva@gmail.com (A.A.M.d.S.); 2 Ribeirão Preto Medical School, University of São Paulo, Ribeirão Preto 14049-900, Brazil; evianna@fmrp.usp.br (E.d.S.O.V.); hbettiol@fmrp.usp.br (H.B.); mabarbieri@fmrp.usp.br (M.A.B.)

**Keywords:** ultra-processed foods, asthma, bronchial hyperreactivity, cross-sectional study

## Abstract

Background: Ultra-processed Food (UPF) consumption can play a role in the pathogenesis and progression of asthma. The aim of this study was to evaluate the association between the consumption of UPF and asthma. Methods: This cross-sectional study included 1857 adults aged 23–25 years from the Ribeirão Preto-SP birth cohort (1978/1979). The exposure variable was the consumption of UPF (expressed as their percentage contribution to energy intake—% total caloric value [%TCV] and their percentage contribution to the amount of food ingested—%grams), which was assessed with a food frequency questionnaire. Asthma was the outcome and was defined based on a positive methacholine challenge test and the presence of wheezing, chest tightness, or shortness of breath over the last 12 months. Poisson regression with robust variance was used to estimate the association between these variables. Unadjusted analyses and analyses adjusted for sex, age, household income, smoking, and physical activity level were performed. Results: The prevalence of asthma in the sample was 13.2%. The mean total consumption of UPF was 37.9 ± 11.2% TCV (corresponding to 35.1 ± 15.1% grams). There was no association between the consumption of UPF and asthma in adults. Conclusion: This study provides no evidence for an association between the consumption of UPF and asthma in young adults.

## 1. Introduction

Asthma is a chronic, treatable noncommunicable disease that affects an estimated 339 million people worldwide [1] and is the second most common chronic respiratory disease [2]. In Brazil, the National Health Survey identified that 5.3% of Brazilians older than 18 years had a diagnosis of asthma [3].

Due to its different underlying etiological processes, asthma is a heterogeneous inflammatory disease that is characterized by inflammation of the airways, airflow obstruction, and bronchial hyperresponsiveness. The clinical definition of asthma is generally based on a history of one or more respiratory symptoms such as wheezing, chest tightness, shortness of breath, and a cough that vary over time and in intensity. Since these symptoms are not limited to this pathological condition and may be confused with other respiratory diseases, available objective methods such as spirometry and the bronchoprovocation test can increase the accuracy when diagnosing asthma [4]. Genetic and epigenetic factors, in combination with exposure to environmental factors are considered strong risk factors, for the development of asthma [5,6].

The diet can influence some factors that play an important role in the etiology of asthma, including the intestinal microbiota, epigenetic modulation, physical development, and airway remodeling [7]. Furthermore, the consumption of proinflammatory diets could favor low-grade systemic and airway inflammation (typical of asthma) through mechanisms that are not yet fully understood [8]. According to the guidelines of the Global Initiative for Asthma, dietary factors are key to the primary prevention of asthma, including a diet that is rich in fruits and vegetables [4].

For a long time, nutrition- and health-related public policies, recommendations, and interventions have conventionally been based on the nutrients present in different types of food and beverages. However, Monteiro et al. [9] drew attention to the fact that this approach focused only on specific nutrients or foods and neglected the effects of processing. In an attempt to describe food systems and eating patterns and how these could affect health and disease risk, Monteiro et al. [10] developed the NOVA food classification. This system was updated in 2012 and divided into four groups: group 1 (unprocessed or minimally processed foods), group 2 (processed culinary ingredients), group 3 (processed foods), and group 4 (ultra-processed foods, UPF).

The ultra-processing of food creates attractive, hyper-palatable, inexpensive, and ready-to-consume products. However, nutritionally, these products are energy-dense, are high in fat, added sugar, or salt, and are generally obesogenic. The practicality and low cost of ultra-processed products have led to an increase in their consumption. In Brazil, studies have indicated the growing presence of UPF in the diet of families. This shift in the Brazilian diet is one of the main causes of the current pandemic of chronic diseases [11].

The growing urbanization that occurs, especially in developing countries, is associated with both an increase in the consumption of UPF and in the prevalence of asthma. Studies have shown that some foods, including nutrients or dietary patterns, as well as the period of exposure (prenatal, childhood, adulthood), play a role in the pathogenesis and progression of asthma [12]. Evidence on the association of processed meat consumption with an increase in asthma symptoms [13], the beneficial effect of fresh fruits and antioxidant vitamins on asthma [14], and the correlation of the adoption of a “Western diet” with poor asthma control [15] highlight the need to investigate whether an association exists between a specific food group according to the degree of processing, especially UPF, and asthma considering the increased consumption of these foods.

A cross-sectional study of young adults with a mean age of 22.8 years identified that UPF accounted for 51.2% of the total energy intake in the population studied [16]. In the study by Juul et al. [17], which analyzed the consumption of UPF in adults over a period from 2001 to 2018 using NHANES data, the consumption of these foods ranged from 53.5% to 57%. Two Brazilian studies involving children and adolescents evaluated the association between UPF consumption and asthma. One study reported an association in adolescents, while the other study found no association between UPF consumption in childhood and asthma in adolescence [18,19]. A study of 513 children demonstrated an 83% increase in the prevalence of wheezing respiratory diseases associated with high consumption of UPF [20]. However, studies on adults conducted in other countries found no association between the consumption of UPF and asthma [21,22].

Considering that both the consumption of UPF and the prevalence of asthma have increased and that epidemiological studies are scarce and their results regarding this association are inconclusive, this study aimed to evaluate the association between the consumption of UPF and asthma in adults using a more accurate diagnosis of asthma based on the presence of symptoms and a positive objective test.

## 2. Methods

### 2.1. Study Design and Sample

This is a cross-sectional study that used data from the project entitled “From perinatal health to the health of young adults: a study of a cohort born in 1978/79 in hospitals of Ribeirão Preto, São Paulo”. The present study is part of the fourth phase conducted from 2 April 2002 to 12 May 2004, when individuals of the original cohort were 23–25 years old.

The baseline data of this cohort were obtained by interviews with 9067 mothers held immediately after childbirth. This number corresponded to 98% of live births that occurred in the eight maternity hospitals in Ribeirão Preto between 1 June 1978 and 31 May 1979. Of these respondents, 2094 were excluded because they were not residents in the city at the time of delivery [23]. A total of 6973 newborns remained in the study; of these, 6827 were single fetuses, and 146 were twins. Multiple births were excluded. Among the 6827 singleton children, 257 died within the first year of life and 86 before the age of 20, totaling 343 deaths.

**Figure 1:**
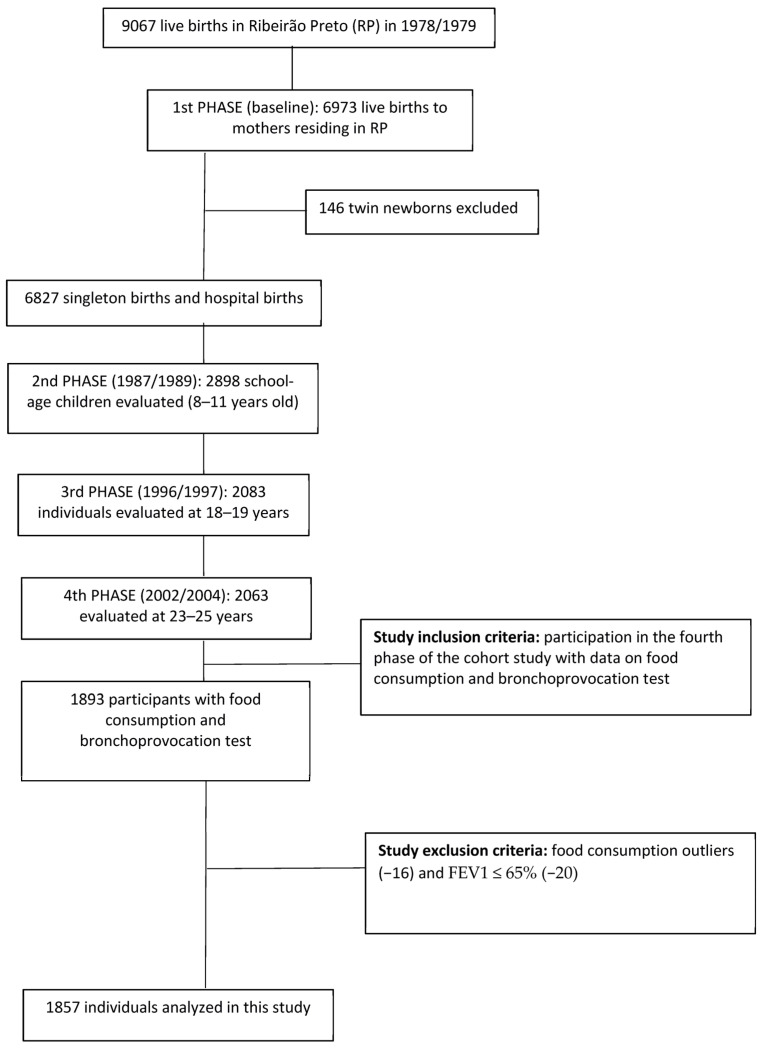
Flow diagram of participants in the final sample of this study on the association between ultra-processed food consumption and asthma in the fourth phase of the Ribeirão Preto-SP birth cohort.

In 2005/2006, when the participants were between 23 and 25 years old, 5665 of the 6484 individuals who lived up to the age of 20 were located through the Hygia system (electronic scheduling system for users of medical services of the Brazilian public health system [SUS]) based on the list of health plan users and the list of children evaluated at school age. Finally, 2103 participants were reassessed. Forty twins were excluded, leaving 2063 individuals [23]. For this study, 29 participants were also excluded because they had no data on the food frequency questionnaire (FFQ), 141 because they did not undergo the bronchoprovocation test, 20 that presented forced expiratory volume in the first second (FEV1) ≤ 65% and 16 because they reported energy intake outside the mean ± 3 standard deviations, considered implausible [24], totaling 1857 individuals in the final sample (Figure 1).

### 2.2. Food Intake and Consumption of Ultraprocessed Foods

The consumption of UPF was the exposure and was expressed as the percentage contribution of UPF energy to the total energy intake and the percentage contribution of the amount of food ingested (grams) of UPF to the total food ingested (grams), which was reported as a continuous variable and in terciles. This variable was obtained by collecting data on food intake over the last 12 months from an FFQ. The FFQ applied in the fourth phase of the cohort was developed and adapted from questionnaires validated in other studies, such as that involving the Japanese–Brazilian community in São Paulo [25]. This questionnaire was later adapted for use in programs for preventing chronic noncommunicable diseases in adults, including the age group studied here [26,27].

The questionnaire consisted of 75 food items, in addition to open spaces so that foods not included but usually consumed by the respondents could be added. For each food, the number of times (with 10 options: 0 to 9 times), the frequency of consumption (daily, weekly, or monthly), and the size of the portion (small, medium, or large corresponding to the 25th, 50th and 75th percentile, respectively) were reported. The questionnaire was applied by nutritionists who used a photo album in order to minimize memory bias and improve the quality of information on portion size [28].

For the calculation of the intake of each food in grams (g) or milliliters (mL), the reported frequencies were transformed into the daily frequency and multiplied by portion size as follows: when the unit of time reported was day, food intake was calculated as N × U × P, where N = the number of times consumed per day, U = 1, and P = the amount of food consumed according to the portion size of each food. When the unit of time was a week, food intake was calculated as N × 1/7 × P, where the weekly frequency was converted to daily frequency by dividing it by the number of days of the week (7). When the unit of time was in months, food intake was calculated as N × 1/30.4375 × P. In this case, the monthly frequency was converted to the daily frequency by dividing it by the number of days in a month, considering 365 days for common years and 366 days for leap years. The constant (30.4375) was obtained as the sum of 3 common years + 1 leap year [3 × 365 + 366], divided by 48, and referred to the total number of months in a four-year period [4 × 12], corresponding to an average of 30.4375 days per month. A value of zero was assigned when the food was not consumed by the participant [29].

Based on the intake of each food, in g or mL, nutrient intake was calculated using food composition tables [30,31,32]. The energy intake from each food was estimated by multiplying carbohydrate and protein values by 4 kcal and lipid values by 9 kcal. The daily energy intake from each food was obtained based on the total calories of each macronutrient. In the case of alcoholic beverages, the kilocalories from alcohol were also considered (7 kcal/g). The total daily energy intake was estimated by summing all consumed calories from all FFQ items. The macronutrient intake was adjusted to 1000 kcal/g.

The foods were grouped according to the NOVA classification into unprocessed or minimally processed foods, processed foods, and UPF. Culinary ingredients were grouped together with unprocessed or minimally processed foods in a group called unprocessed foods/culinary preparations [33]. When foods from different groups were grouped in the FFQ, we chose to divide the participation of these foods into more than one group using the consumption observed in the state of São Paulo as a parameter according to the annual Household Budget Survey (2002–2003) [34]. For example, in the case of the food item “honey and jam”, 65% was allocated to the group of unprocessed or minimally processed foods (related to honey), and 35% was allocated to the group of processed foods (in relation to jelly). The percentage contribution of each food group and UPF subgroup to the total dietary energy intake was then calculated.

### 2.3. Asthma

Current asthma symptoms were investigated using a questionnaire that consisted of questions from the European Community Respiratory Health Survey (ECHRS). This questionnaire was applied by a trained physical therapist. The following questions were used: Have you experienced wheezing or whistling in your chest at any time in the last 12 months? Have you woken up with a feeling of tightness in your chest at any time in the last 12 months? Have you experienced shortness of breath during the day when you were at rest at any time in the last 12 months? Have you been woken up during the night by shortness of breath in the last 12 months?

The bronchoprovocation test (methacholine challenge test) was performed in the Pneumology Laboratory of the University Hospital, Ribeirão Preto Medical School, University of São Paulo (HCFMRP-USP), by trained technicians and doctors. For the test, methacholine chloride (Sigma, St. Louis, MO, USA) was diluted in phosphate-buffered saline (PBS) following international references. Individuals with upper respiratory infections, individuals using antihistamines and antibiotics, and pregnant and breastfeeding women were not submitted to the test [35].

First, the participants inhaled 3 mL of a control solution (PBS) for 2 min, and the FEV1 was then measured. As a safety measure, if FEV1 was ≤65%, the test was not started and the participant was excluded because we were unable to obtain evidence of bronchial reactivity. Individuals with FEV1 > 65% were submitted to different concentrations of methacholine, with each concentration being twice the previous one: 0.06, 0.125, 0.25, 0.5, 1.0, 2.0, 4.0, 8.0 and 16 mg/mL. The inhalation procedure of the different concentrations was the same as that of the control solution and was followed by the measurement of FEV1. The inhalations were performed successively until a decline in FEV1 ≥ 20% or until symptoms such as coughing, wheezing, and chest tightness became relevant so that the test had to be interrupted or once a methacholine concentration of 16 mg/mL was reached. Three maneuvers were used to define the decline in FEV1 [35,36]. A dose–response curve was constructed, and the concentration necessary for a 20% decline in FEV1 was calculated.

Bronchial responsiveness to methacholine was defined as normal for concentrations > 8 mg/mL and positive for concentrations ≤ 8 mg/mL. Individuals with a positive bronchoprovocation test and who exhibited any of the self-reported clinical signs were classified as asthmatic [35].

### 2.4. Confounding Variables

A theoretical model of causality based on a directed acyclic graph (DAG) was built using the online DAGitty 3.2 software. The exposure (UPF), outcome (asthma), and confounding variables were included, and causal connections between the variables were established and indicated by arrows.

A minimum adjustment set of variables to control for confounding was selected using the backdoor criterion [37] in order to avoid unnecessary adjustments, spurious associations, and estimation errors. The backdoor criterion considers the need for adjusting for variables that are common causes of exposure and the outcome and for descendants of confounding variables. Adjustments were not suggested for mediating variables (which could block the causal flow and suppress the effect of exposure on this outcome), for colliders (which could lead to bias since colliders block the flow of spurious associations between two variables), and for the descendants of colliders [38].

**Figure 2:**
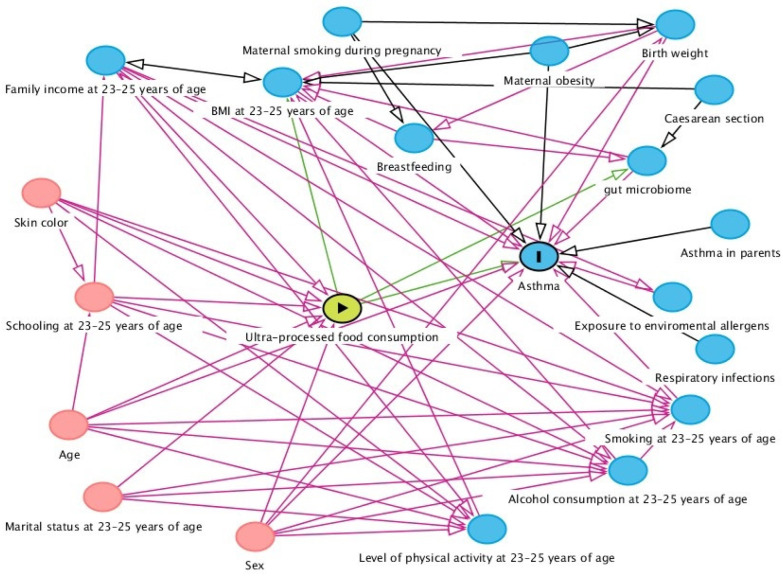
Directed acyclic graph showing the association between the consumption of ultra-processed foods (UPF) and asthma.

After the analysis of the theoretical model by applying the backdoor criterion, the variables suggested for the minimum adjustment set were sex (male and female), age (23, 24, and 25 years), household income (<5, 5–9.9, >9.9 minimum wages, no information), smoking (yes and no), the level of physical activity (high, moderate, and low), and alcohol consumption. Since the analysis of the FFQ already considered alcohol consumption, it was not used as an adjustment variable. The total caloric value of the diet was added in the analysis of %TCV and %grams (Figure 2). Data referring to the social, demographic, and lifestyle characteristics of individuals aged 23–25 years were obtained using structured questionnaires. Family income was assessed based on the value of the national minimum wage in 2002/2004, and the assessment of smoking was based on the question: do you currently smoke (at least one month ago)? The level of physical activity was assessed according to the International Physical Activity Questionnaire–IPAQ [39].

### 2.5. Statistical Analysis

Data were analyzed using the STATA^®^ 14.0 statistical program. For descriptive analysis, categorical variables were reported as absolute and relative frequencies, and continuous variables as the mean and standard deviation.

Sociodemographic and lifestyle variables were compared between asthmatic and non-asthmatic individuals using Pearson’s chi-square test or Fisher’s exact test. The mean food intake values considering the total intake of calories, macronutrients, and food groups and subgroups were compared between asthmatic and non-asthmatic individuals using Student’s *t*-test. The level of significance was set at 0.05, and a 95% confidence interval was adopted.

To evaluate the association between the percent of energy intake from UPF and asthma, Poisson regression with robust variance estimation was performed since this was a cross-sectional study in which the prevalence of asthma was ≥10% [40]. The unadjusted and adjusted analyses of confounders indicated by the backdoor criterion were performed, excluding alcohol consumption, and the addition of TCV. The analyses included linear variables of UPF consumption and quadratic terms were subsequently added to investigate the possible non-linear effects. The presence of possible interactions (UPF consumption and sex) was also tested. A level of significance of 0.10 was adopted for the interaction test.

This study was approved by the Research Ethics Committee of HCFMRP-USP (Opinion number 7606/99), and all participants signed the free informed consent form.

## 3. Results

There was a predominance of individuals aged 24 years (50.1%) and women (51.8%) in the population studied. Most participants earned less than five minimum wages (32.6%), had a high level of physical activity (47.0%), and were non-smokers (83.4%). The prevalence of asthma was 13.2% in the population studied, with a higher prevalence among women (17.1%) compared to men (9.1%) (Table 1).

The mean (±standard deviation) food intake was 2135.4 g (±751.8 g), with 35.1% (±15.1) of UPF, 7.3% (±5.6%) of processed foods, and 57.6% (±15.2%) of unprocessed foods/culinary preparations (Table 2).

Sugar-sweetened beverages accounted for 23.8% grams of UPF consumed per day and were the most consumed subgroup in the sample studied. In addition to sugary drinks, savory snacks, sweets, and dairy products were the most consumed UPF subgroups in %grams. The percentage contributions of UPF to the total dietary intake (in %grams and %TCV) are shown in Table 3.

The consumption of UPF, expressed as %TCV or %grams, was not associated with asthma in adults (Table 4). There was no interaction between sex and UPF consumption. We also did not find statistical significance in the analysis using quadratic terms of UPF consumption (in %grams and %TCV) when investigating the non-linear effects.

## 4. Discussion

In the present study, neither UPF consumption expressed as %TCV nor as %grams (continuous and tertiles) were significantly associated with asthma in adults aged 23–25 years. The consumption of UPF accounted for 37.9% and 35.1% of the total energy intake and the total dietary intake in grams of the participants in the present study, respectively. Other studies involving Brazilian individuals of a similar age range have reported divergent results. Bielemann et al. [16] found a caloric contribution of 51.2% in this food group among 23-year-old adults from the 1982 Pelotas cohort. On the other hand, in the study by Costa et al. [41], the energy contribution of UPF was 29.6% among 22-year-old participants in the 1993 Pelotas cohort. Regarding the contribution of UPF to the amount of food ingested, Silva et al. [42] reported a percentage of UPF in grams of 9.2% in a sample of 506 individuals aged 20 years or older living in the city of Brasília.

With respect to the diagnosis of asthma, our study found a higher prevalence of this respiratory disease at 23–25 years of age (13.2%) than that reported in studies involving individuals aged 18 to 29 years in Brazil [43] and in the world [2]. When the prevalence was estimated by gender, the results were similar to global and national data reported in the literature, which show a higher prevalence of asthma in females in this age group [2,43]. The higher prevalence of asthma observed in this study compared to other investigations can probably be related to differences in the diagnostic methods used. In the present study, asthma was defined based on a positive bronchoprovocation test with methacholine and the presence of different clinical symptoms, whereas most studies in the literature, including those cited above, used only self-reported wheezing in the last 12 months and a medical diagnosis of asthma for the definition of asthmatic individuals. The combination of the presence of self-reported clinical signs and an objective method, such as the positive methacholine challenge test used in our study, improved the identification of individuals with asthma, reducing false-positive results. Although the indicator of airflow obstruction through wheezing may not be an exclusive symptom of asthmatic patients, a negative bronchoprovocation test in symptomatic individuals ruled out the diagnosis of asthma as a cause of this symptom [44], resulting in a lower prevalence of the disease. However, it must be noted that symptoms other than wheezing were evaluated in the present study, a fact that could explain the observation of a higher prevalence of asthma. Furthermore, the self-reported medical diagnosis of asthma used in other studies is prone to memory bias and is influenced by the limited access of individuals to health services [2]: factors that could underestimate the prevalence in these studies when compared to other studies involving the same age group.

Regarding the observation of a higher prevalence of asthma among women, the literature reports that asthma becomes more prevalent and severe in females after puberty, with a higher prevalence among women with early menarche and those with multiple pregnancies. These findings suggest the role of sex hormones in the development of asthma. However, the effect of sex hormones on the pathophysiology of asthma can be mediated by obesity, atopy, and other gender-associated environmental exposures. Furthermore, gender differences in the perception of asthma symptoms have been reported, which could also affect the prevalence estimates of the disease [45]. Although not the aim of this study, the combination of hormonal and environmental factors, nutritional status, other allergic manifestations, and the perception of symptoms seemed to increase the risk of developing the disease and the probability of its detection in women, which could explain the higher prevalence of asthma among the women observed here.

Most studies investigating the association between the consumption of UPF and asthma have been conducted with children and adolescents, and the results are divergent [18,19]. In the study by Melo et al. [19], in which asthma was defined based on an affirmative response to the questions “Have you had wheezing or chest tightness in the last 12 months?” and “Have you ever had asthma?” the consumption of UPF was positively associated with the presence of asthma in Brazilian adolescents. The adjusted odds ratio (OR) for asthma was 1.27 (95% CI: 1.15–1.41) when the highest and the lowest quantiles of the UPF consumption score were compared. Azeredo et al. [18] defined asthma based on the parental reporting of medical diagnosis or wheezing in the last 12 months. However, the authors found no association between the consumption of UPF and asthma in childhood or adolescence. At 6 years of age, the adjusted OR comparing children in the lowest and highest quantiles of UPF consumption was 0.84 (95% CI: 0.28–1.21) for asthma and 1.12 (95% CI: 0.62–2.03) for severe asthma, whereas at 11 years the adjusted OR for asthma and severe asthma was 1.00 (95% CI: 0.70–1.44) and 1.05 (95% CI: 0.59–1.86), respectively. Li et al. [21] also found no association between the consumption of UPF and asthma in adults. This definition of asthma was defined based on the medical and death records of the UK Biobank. In individuals aged 40 to 69 years, the adjusted hazard ratio comparing individuals in the lowest and highest quartile of UPF consumption was 1.03 (95% CI: 0.89–1.18).

Some plausible biological mechanisms have been suggested to explain the apparent association between the higher consumption of UPF and asthma. These foods generally contain high levels of free sugars, total saturated and trans fats, salt, preservatives, and other food additives, which could trigger reactions in the body that favor the development of asthma [46]. The dietary fatty acid composition can modulate immune reactions by regulating T helper 2 cell-mediated (pro-allergic) immune responses, and a diet that is high in saturated fats and cholesterol can exacerbate this immune response, leading to the inflammation of the airways [47]. Preservatives such as sodium benzoate or sulfites, which are present in soft drinks and other UPF, have been associated with the aggravation of asthma symptoms [48,49]. Furthermore, nitrite, which occurs at high levels in cured meats, can cause the stress-related nitrosative inflammation of the airways that is involved in asthma [50].

The present study has some limitations. One limitation is related to the cross-sectional design of this study. Since the food consumption data refer to the last 12 months and asthma was defined based on the self-report of clinical symptoms over the last year, although the bronchoprovocation test expressed current bronchial responsiveness, it was not possible to establish the temporality between exposure and outcomes. Another limitation was related to the method used for measuring exposure, which was prone to memory and self-report bias. To minimize this bias, the interviewers were trained in order to ensure the proper application of the questionnaire, and a photo album was used to measure portion size.

Food frequency questionnaires are widely used in epidemiological studies for assessing habitual dietary intake and do not change the pattern of food consumption when methodological parameters are carefully planned, thus ensuring the reliability and accuracy of the data [51]. However, the use of a FFQ was a limiting factor in our study since it was not originally designed to identify food intake according to the degree of processing, considering that the NOVA classification was developed after the elaboration of this instrument. We, therefore, grouped some foods that belonged to different groups according to the NOVA classification in the same food item. To overcome this limitation, we made some adjustments considering the food intake estimates for the State of São Paulo at the time of data collection [34]. In addition, the FFQ was adapted for use in programs to prevent chronic noncommunicable diseases in adults, reflecting food intake at the time.

On the other hand, this study has several strengths. To our knowledge, this is the first study that investigates the association between the consumption of UPF and asthma in young adults. Although asthma is more frequently investigated in the pediatric population, it continues to be an important respiratory problem with increasing age and after puberty, which could be linked to lifestyle factors such as higher consumption of UPF in adult life. Furthermore, the definition of asthma based on clinical symptoms combined with the objective measurement of lung function by the bronchoprovocation test reduced the possibility of classification errors in the outcome (false positives).

## 5. Conclusions

In conclusion, the present study provides no evidence for an association between the consumption of UPF and asthma in young adults.

## Figures and Tables

**Table 1:** Sociodemographic and lifestyle variables of adults (23–25 years) with or without asthma in the fourth follow-up of the Ribeirão Preto birth cohort (1978/1979), São Paulo, Brazil (2002–2004).

Variables	Total	Asthma *		
	*n* (%)	No (*n*, %)	Yes (*n*, %)	*p*-Value #
Sex				<0.001
Male	895 (48.2)	814 (90.9)	81 (9.1)	
Female	962 (51.8)	798 (82.9)	164 (17.1)	
Age				0.008
23 years	521 (28.0)	432 (82.9)	89 (17.1)	
24 years	930 (50.1)	821 (88.3)	109 (11.7)	
25 years	406 (21.9)	359 (88.4)	47 (11.6)	
Household income **				0.002
<5 MW	605 (32.6)	504(83.3)	101 (16.7)	
5–9.9 MW	568 (30.6)	499 (87.8)	69 (12.2)	
>9.9 MW	553 (29.8)	500 (90.4)	53 (9.6)	
No information	131 (7.0)	109 (83.2)	22 (16.8)	
Physical activity level				0.094
High	873 (47.0)	767 (87.9)	106 (12.1)	
Moderate	590 (31.7)	518 (87.8)	72 (12.2)	
Low	389 (21.0)	323 (83.0)	66 (17.0)	
No information	5 (0.3)	4 (80.0)	1 (20.0)	
Smoking				0.001
No	1548 (83.4)	1360 (87.9)	188 (12.1)	
Yes	309 (16.6)	252 (81.6)	57 (18.4)	
Total	1857 (100.0)	1612 (86.8)	245 (13.2)	

* A positive bronchoprovocation test and the presence of clinical signs (wheezing in the last 12 months or chest tightness in the last 12 months or shortness of breath during the day. when at rest in the last 12 months or shortness of breath at night in the last 12 months). ** MW, minimum wage ($66.00 to 86.00 from 2002 to 2004). # Pearson’s chi-squared test or Fisher’s exact test.

**Table 2:** Contribution of food groups and macronutrients to the total energy and dietary intake of adults (23–25 years) from the fourth follow-up of the Ribeirão Preto birth cohort (1978/1979), São Paulo, Brazil (2002–2004).

Food Groups		Total (*n* = 1857)		Asthma				*p*-Value
				No (*n* = 1612)		Yes (*n* = 245)		
		Mean	SD	Mean	SD	Mean	SD	
Total energy kcal/day		2237.3	705.8	2232.3	701.8	2270.1	732.0	0.435
Total intake		2135.4	751.8	2132.9	761.0	2152.0	695.3	0.712
Unprocessed/minimally processed	kcal of diet	1120.0	380.2	1120.0	379.0	1118.5	388.2	0.961
	%TCV	50.9	11.2	51.1	11.3	50.1	11.0	0.196
	g/day	1191.5	444.5	1190.5	446.1	1198.2	435.0	0.802
	%g/day	57.6	15.2	57.6	15.3	56.9	14.4	0.502
Processed	kcal of diet	246.0	142.4	244.0	139.0	257.3	163.0	0.167
	%TCV	11.0	5.4	11.0	5.2	11.2	6.1	0.455
	g/day	155.6	136.3	153.9	133.9	166.6	151.0	0.177
	%g/day	7.3	5.6	7.3	5.5	7.6	6.3	0.372
Ultraprocessed	kcal of diet	869.3	434.1	866.0	433.3	892.0	439.3	0.386
	%TCV	37.9	11.2	37.8	11.3	38.6	10.8	0.333
	g/day	788.3	580.0	788.5	593.0	787.2	487.2	0.975
	%g/day	35.1	15.1	35.0	15.2	35.4	15.2	0.729
Macronutrients and alcohol								
Carbohydrates	%TCV	55.7	6.7	55.8	6.6	55.5	6.8	0.544
	g/1000 kcal	139.4	16.6	139.5	16.6	138.8	17.0	
Proteins	%TCV	16.8	3.2	16.9	3.2	16.3	3.2	0.008
	g/1000 kcal	42.1	7.9	42.3	7.9	40.8	7.9	
Lipids	%TCV	26.1	4.9	26.0	4.8	26.7	5.1	0.037
	g/1000 kcal	29.0	5.4	28.9	5.4	29.7	5.7	
Fibers	g/1000 kcal	11.1	3.0	11.1	3.0	11.0	2.8	0.704
Alcohol	%TCV	1.3	1.8	1.3	1.7	1.4	2.3	0.215
	g/1000 kcal	1.8	2.6	1.8	2.5	2.0	3.2	

TCV: total caloric value; SD: standard deviation.

**Table 3:** Contribution of ultra-processed foods by subgroup to the total energy and dietary intake of adults (23–25 years) from the fourth follow-up of the Ribeirão Preto birth cohort (1978/1979), São Paulo, Brazil (2002–2004).

Ultraprocessed Foods	Total		Asthma				*p*-Value *
			No		Yes		
	Mean	SD	Mean	SD	Mean	SD	
%TCV							
Sugar-sweetened beverages	9.9	7.8	9.9	8.0	9.8	7.0	0.900
Savory snacks	8.2	7.0	8.2	7.0	7.5	6.3	0.124
Sweets/desserts	4.4	4.0	4.4	4.0	4.4	4.0	0.803
Cookies	3.0	3.2	2.9	3.1	3.4	3.9	0.012
Breads	2.1	2.0	2.1	2.0	2.0	2.0	0.380
Sausages	1.9	1.8	1.9	1.7	2.0	1.9	0.213
Cakes	1.9	2.6	1.9	2.4	2.3	3.6	0.013
Chips	1.7	2.6	1.7	2.6	2.2	2.8	0.005
Dairy products	1.6	2.1	1.6	2.1	1.8	2.2	0.223
Ramen noodles	1.0	0.8	1.0	0.8	1.0	0.7	0.679
Margarine	0.8	1.1	0.8	1.1	0.9	1.0	0.279
Granola	0.6	1.6	0.6	1.6	0.3	1.0	0.008
Mayonnaise	0.5	0.8	0.5	0.8	0.6	1.1	0.597
Hard liquors	0.1	0.6	0.2	0.6	0.1	0.5	0.442
%grams							
Sugar-sweetened beverages	23.8	15.0	23.7	15.1	24.0	14.1	0.918
Savory snacks	3.5	3.4	3.6	3.4	3.3	3.0	0.164
Sweets/desserts	1.4	1.3	1.4	1.3	1.4	1.3	0.621
Dairy products	1.4	1.9	1.4	1.9	1.5	1.9	0.494
Sausages	1.1	1.0	1.1	0.9	1.2	1.1	0.199
Cakes	0.9	1.2	0.8	1.1	1.0	1.7	0.013
Breads	0.8	0.8	0.8	0.8	0.8	0.8	0.591
Cookies	0.7	0.8	0.7	0.8	0.8	1.0	0.026
Chips	0.4	0.7	0.4	0.7	0.5	0.7	0.030
Ramen noodles	0.4	0.4	0.4	0.4	0.4	0.3	0.705
Granola	0.2	0.4	0.2	0.5	0.1	0.3	0.015
Mayonnaise	0.2	0.3	0.2	0.3	0.2	0.5	0.296
Margarine	0.1	0.2	0.1	0.2	0.2	0.2	0.310
Hard liquors	0.1	0.2	0.1	0.2	0.1	0.2	0.496

TCV: total caloric value; SD: standard deviation. * Student *t*-test.

**Table 4:** Association between the consumption of ultra-processed foods and asthma in adults (23–25 years) from the fourth follow-up of the Ribeirão Preto birth cohort (1978/1979), São Paulo, Brazil (2002–2004).

Unadjusted Analysis				Adjusted Analysis ^a,b^	
Ultraprocessed Food Consumption	*n*	PR (95% CI)	*p*-Value	PR (95% CI)	*p*-Value
Tertile (% kcal)			0.932 ^c^		0.928 ^c^
1st tertile	619	Reference	-	Reference	-
2nd tertile	619	1.17 (0.88–1.55)	0.280	1.19 (0.90–1.58)	0.212
3rd tertile	619	1.01 (0.75–1.36)	0.932	0.99 (0.74–1.32)	0.935
% kcal	1857	1.00 (0.99–1.01)	0.315	1.00 (0.99–1.01)	0.466
Tertile (% g)			0.934 ^c^		0.942 ^c^
1st tertile	619	Reference	-	Reference	-
2nd tertile	619	0.94 (0.70–1.25)	0.673	0.98 (0.74–1.32)	0.925
3rd tertile	619	1.01 (0.76–1.34)	0.934	0.99 (0.73–1.32)	0.942
% g	1857	1.00 (0.99–1.00)	0.714	1.00 (0.99–1.00)	0.937

PR: prevalence ratio; 95% CI: 95% confidence interval. ^a^ Analysis of % energy adjusted for the variables age, physical activity level, sex, income, and smoking and for kilocalories. ^b^ Analysis of % grams adjusted for the variables age, physical activity level, sex, income, and smoking and for kilocalories. ^c^ *p*-value for trend.

## Data Availability

The data that support the findings of this study are available on request from the corresponding author. These data are not publicly available due to privacy or ethical restrictions.
